# Records of *Coendou ichillus* (Rodentia, Erethizontidae) from the Lower Urubamba Region of Peru

**DOI:** 10.3897/zookeys.509.9821

**Published:** 2015-06-24

**Authors:** Tremaine Gregory, Darrin Lunde, Hugo Tomás Zamora-Meza, Farah Carrasco-Rueda

**Affiliations:** 1 Center for Conservation Education and Sustainability, Smithsonian Conservation Biology Institute, National Zoological Park, Washington, DC 20013-7012; 2 Department of Vertebrate Zoology, Division of Mammals, National Museum of Natural History, Washington, DC 20013-7012; 3 Museo de Historia Natural Universidad Nacional de San Agustín, Arequipa, Peru; 4 School for Natural Resources and Environment, University of Florida, Gainesville, FL 32611

**Keywords:** Neotropical porcupines, *Coendou ichillus*, Peru, biodiversity, distribution, Urubamba, Rodentia, camera trap

## Abstract

*Coendou ichillus* was first described in 2001 by Voss and da Silva, with a range from Amazonian Ecuador to Iquitos, Peru. Here, we describe an adult female *Coendou ichillus* specimen collected in a Tomahawk trap in the forest canopy of the Lower Urubamba Region of Peru in October 2013. We also describe pathologies and behaviors observed through 379 camera trapping photo events (2,196 photos) gathered in natural canopy bridges over the course of a year (7,198 trap nights), including information on activity period over the course of the day and over the course of the lunar cycle. We conservatively estimate that 17 individuals were photographed, including one juvenile. Being 900 km away from Iquitos, Peru (the site of the closest record), discovery of this species in the Lower Urubamba constitutes a significant range extension.

## Introduction

Our understanding of the diversity and distributions of Neotropical porcupines of the *vestitus*-group has been very much thwarted by the lack of specimens and locality records. Voss and da Silva (2001) discussed two hypotheses for why these diminutive species might be so rarely detected: 1) they might have very restricted distributions in western Amazonia, and 2) their apparent rarity might be an artifact of inadequate collection. In a recent methods paper, Gregory et al. (2014) demonstrated how specialized camera trapping methods can result in the documentation of a greater abundance of *vestitus*-group porcupines than might have been detected using conventional survey methods (*sensu* Voss and Emmons 1996) alone. Testing either of these hypotheses would begin with efforts to first map the known distribution of the species through the steady accumulation of point locality data. In this paper we report one such new locality record—and a significant range extension—for the recently described species *Coendou ichillus*, which is here reported from the Lower Urubamba Region (LUR) of Peru.

*Coendou ichillus* was previously known from six sight records and only five specimens—three collected from the Amazonian lowlands of eastern Ecuador, one from Iquitos, Peru, and the fifth of unverified natural origins that was purchased in a market in Iquitos (Voss 2011; Voss and da Silva 2001; Voss et al. 2013). The skulls of the specimens analyzed by Voss and da Silva (2001) exhibit damage that presumably occurred during the process of collecting and preparing these specimens, and none include postcranial elements beyond those left in the hands and feet of their respective study skins. Morphometric information was not provided for the specimen from Iquitos mentioned by Voss (2011) and Voss et al. (2013) (TTU 115491). The specimen that forms the basis of our new record consists of a study skin, a skeleton, and an intact skull. To maximize the utility of the specimen, we cleaned the skeletal elements from one side of the study skin, while leaving the other side intact as a traditional study skin. We provide measurements and descriptions of these and compare them to the previously known specimens reported in Voss and da Silva (2001).

In 2011, Gregory and colleagues began a study in the Lower Urubamba Region (LUR) of Peru assessing the effectiveness of natural canopy bridges (connections between the branches of large trees) over a ~15-meter-wide natural gas pipeline clearing in mitigating the ensuing canopy fragmentation. The study began before the natural bridges were exposed and continued through pipeline right-of-way (RoW) construction, revegetation, and eventual closure of the RoW to motorized vehicles. In September 2012, soon after the 13 natural bridges were exposed in July, camera traps were placed in the canopy at all potential crossing points. The 24 cameras were left for a year, and the photos revealed extensive small and medium-size mammal activity in the canopy, including thousands of photos of a dwarf porcupine species. With no dwarf porcupine species known to the area, the research team opted to attempt trapping a specimen for identification as the study concluded. One female specimen was collected.

In this paper we report the range extension for this species, describe its morphology, comment on its behavior as revealed in the camera trap photos, and discuss the significance of the range extension. This is the second in a series of four papers that address the methods used and results from the three-year-long study (Gregory et al. 2014).

## Methods

**Figure 1.**
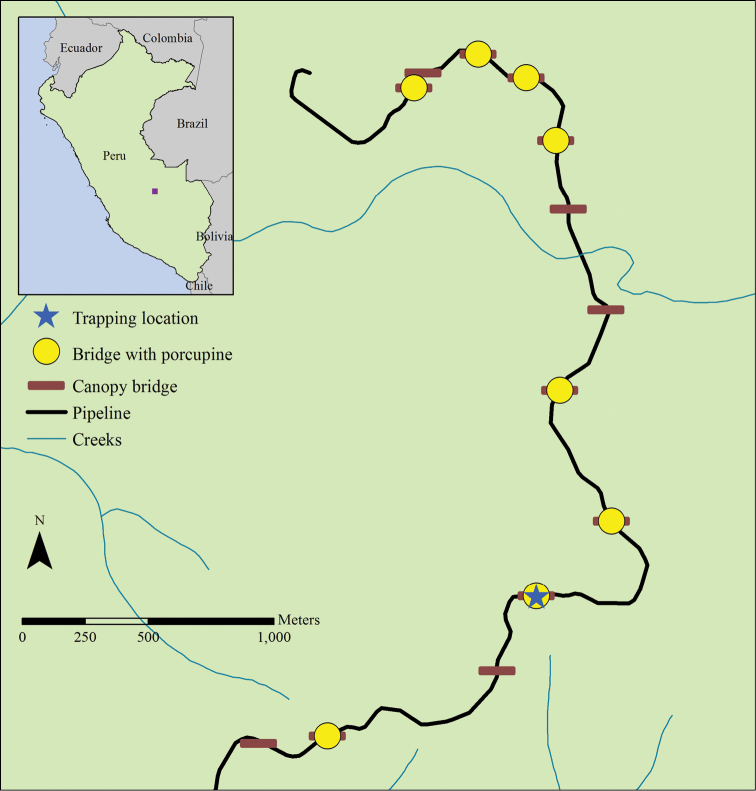
Map of trapping locations and canopy bridge distribution. Yellow circles indicate canopy bridges in which *Coendou ichillus* was photographed with a camera trap. The blue star indicates the bridge in which the specimen was trapped.

The study site is in the Lower Urubamba Region, adjacent to the confluence of the Camisea and Urubamba Rivers (11°43.28'S, 72°56.52'W (DDM), Figure 1). The study area is in predominantly primary forest between 450–500 m with three major habitat types: *terra firme*, riverine terrace, and mixed upland (Comiskey et al. 2001). It is near the Pluspetrol-controlled Pagoreni A natural gas well and the pipeline that connects the well to the Malvinas processing plant. The site is also near the Matsigenka communities of Camisea and Shivankoreni and is not part of the national system of protected areas, for which reason subsistence hunting of wildlife is legal.

This study is part of a larger study aimed at understanding the utility of natural canopy bridges in reducing the forest fragmentation impact caused by a natural gas pipeline right-of-way (RoW) clearing. In order to evaluate natural bridge use by arboreal mammals, the 13 bridges were monitored with 24 Reconyx PC800 Hyperfire^TM^ Professional (Reconyx Inc., Holmen, WI, USA) camera traps placed at all potential crossing points over the course of a year. An additional 28 camera traps were placed on the ground below the bridges and 26 more in a control area along the RoW with no canopy bridges. The branches comprising the bridges were left in July 2012, and monitoring occurred from September 2012, the middle of the pipeline construction period, to September/October 2013, approximately six months post construction. All behavioral data presented here were gathered from camera trap photos. Camera trap photographs were processed by a team of three people who separated the photos into trigger events and identified the vertebrates in each event. The information recorded for each event included the following: date and time, bridge, species present, number of individuals, behaviors exhibited, and moon phase.

To evaluate activity hours of *Coendou ichillus*, data from all cameras over the course of the year were pooled by hour of the day. For the moon phase analysis, data from all cameras were pooled by moon phase for each lunar cycle over the course of 13 cycles. Waning and waxing moon phases with similar amounts of moonlight were pooled for the analysis for a total of 5 moon phase categories: 0 = new moon, 1 = waxing and waning crescent, 2 = first and last quarters, 3 = waxing and waning gibbous, and 4 = full moon. The number of events per hour of the day and per moon phase category per lunar cycle were divided by the corresponding number of camera trap nights and multiplied by 100, for analysis of event rates. A Spearman’s correlation was performed on the moon phase data for the 13 lunar cycles through which the study occurred to explore the relationship between activity and phase.

Regarding other local fauna besides *Coendou ichillus*, the camera trap photos have recorded 20 other mammal species in the canopy, with an additional two species of monkeys registered through visual surveys, and cameras on the ground recorded 22 mammal species (5 species overlapped between the canopy and the ground) (Gregory et al. 2012; Gregory et al. 2014).

Porcupine specimen collection took place in October 2013. Six Tomahawk (20 × 60 × 20 cm) traps were placed in the mid-canopy over 12 days (trapping methods will be described in detail in Gregory, in prep.). Each Tomahawk trap was monitored with a camera trap.

**Figure 2.**
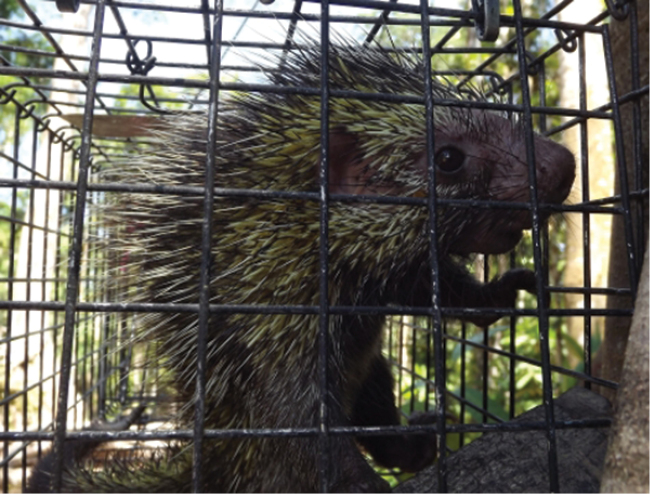
Adult female *Coendou ichillus* (CORBIDI-MA-00973) specimen in life.

The specimen described here was captured on the first day of trapping at 2:26AM (determined by camera trap photo) in a trap at 17.5 m in height (Figure 2). The trap had been baited with a section of wood soaked in salt water and a mixture of canned tuna, oatmeal, tomato paste, and fresh tomato. There was no sign of any remaining bait when the individual was captured. The camera trap documented the individual consuming the bait.

The morning after the specimen was trapped, it was immediately transported in the trap back to the research camp. The entire trap was placed in a large plastic bag with isoflurane-soaked cotton in one corner, out of contact with the animal. After the animal was subdued, it was injected with sodium pentobarbital (6 mL injected into the chest and 2 mL injected into the cranium). The specimen was identified as a female with developed, turgid nipples and a closed vagina. The skin was then prepared and the body was placed in alcohol. Tissue samples, quills, fleas, feces, and stomach contents were also collected. The specimen was deposited at the Centro de Biología y Biodiversidad (CORBIDI, MA-00973) in Lima, Peru, and then borrowed and transported to the United States. Maceration and cleaning of the carcass and cranium and morphometric analyses were performed at the National Museum of Natural History (NMNH). A portion of each tissue sample was deposited at NMNH (USNM 599500).

All reported measurements are in millimeters (mm). External measurements of the specimen that constitutes our new record are those that were recorded in the field by the collector. Cranio-dental measurements are those defined by Voss and da Silva (2001); long-bone measurements are greatest total length. All osteological measurements were recorded to the nearest 0.1 mm using digital calipers at NMNH.

## Results and discussion

*Coendou ichillus* was recorded by the camera traps in nine of the 13 natural canopy bridges. Behavioral inferences drawn from the photos (i.e. animals searching for the best branch by which to cross) and data recorded on the characteristics of the bridges suggest that the only bridges not used were those without direct contact between the branches that composed the bridge. Lack of saltatory ability is therefore likely to have prevented use of these bridges.

**Figure 3.**
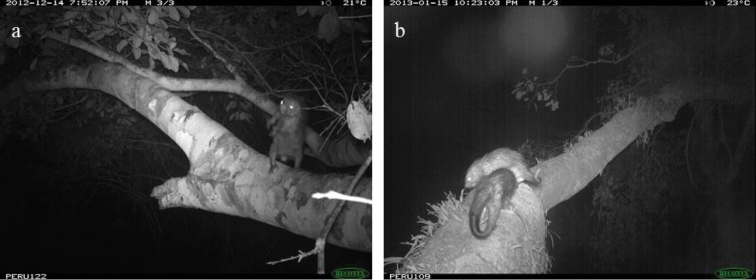
Behaviors demonstrated by *Coendou ichillus*: body shaking (**a**) and body contact between two individuals (**b**).

There were 379 total camera trapping events (2,196 photos) of *Coendou ichillus* over 7,198 trap nights for a rate of 5.3 events/100 trap nights. To identify the number of individuals captured during these events, we carefully analyzed the photos, using identifying characteristics to distinguish between individuals. Our count of the total number of individuals was conservative, accounting for the possibility that close proximity between bridges and favorable topography would allow individuals to be photographed in more than one bridge. In total, we counted 17 individuals, including one juvenile. While adult males and females were not distinguishable, during 11 of the trapping events, two individuals were recorded together. During five of these events (all in the same bridge), one individual was significantly smaller than the other, suggesting it was a mother and juvenile. During the other six events (in two bridges), both animals seemed to be adults and may have been a male and female (Figures 3b and 7c).

Pathologies recorded in the photographs include botfly wounds or lumps on the right and left rear paws and a broken tail. In addition, two individuals showed an enlarged abdomen and may have been pregnant (1 photo event in May, 3 events in July, and 6 events in August). Behaviors recorded include body shaking (N=4 events, Figure 3a), auto-grooming (N=3), pilo-erection (N=42), biting the tree trunk (N=2), and sniffing the tree trunk (N=8), and social behaviors recorded include one individual following the other (N=11) and body contact between individuals (N=2, Figure 3b). Four of the camera traps were heavily gnawed on by *Coendou ichillus* (evidenced by photos); the latches were also opened causing the cameras to flood with rain water.

**Figure 4.**
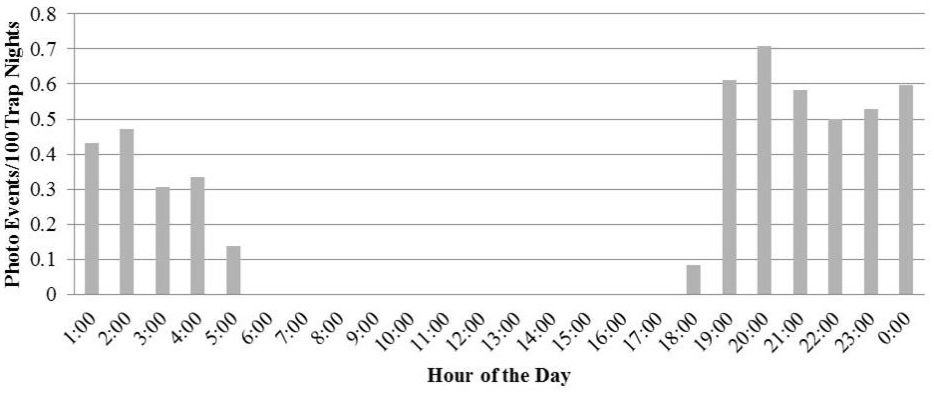
Daily activity pattern of *Coendou ichillus* evaluated through photo events per 100 trap nights over the course of one year.

**Figure 5.**
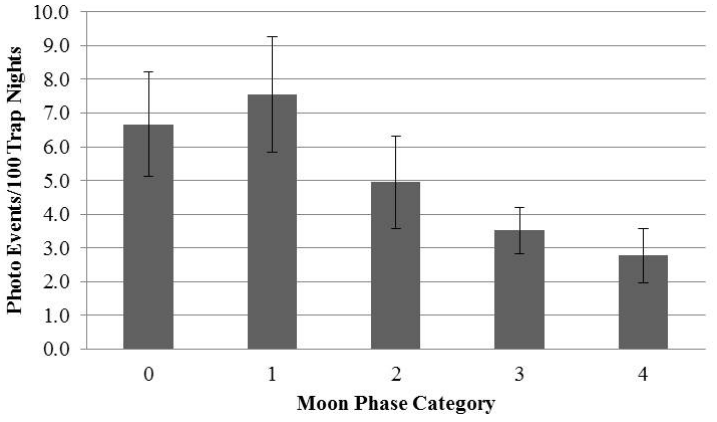
Activity pattern in relation to moon phase evaluated through photo events per 100 trap nights during each moon phase over the course of 13 lunar cycles. Moon phase categories are labeled in order of increasing moonlight (0 = new moon and 4 = full moon). Moon phase categories 1–3 represent data pooled for waning and waxing periods with similar moonlight (e.g. 1 = waxing and waning crescent events pooled). Bars represent the standard error of the mean.

Events of *Coendou ichillus* occurred between 18:00 and 5:00 hours, with an activity peak at 20:00 (Figure 4). With regards to moon phase, there was a significant relationship between activity and phase, and there was more activity during the lower light phases (Figure 5, *ρ* (4)=-0.33, p=0.007).

**Figure 6.**
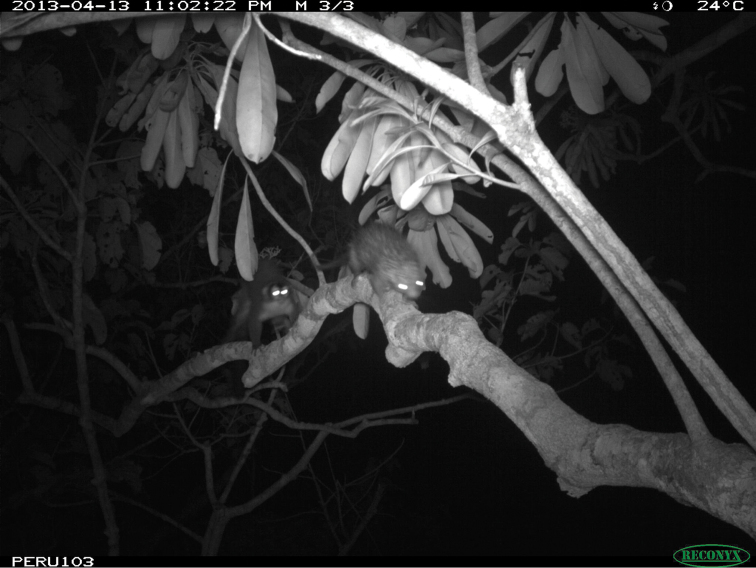
Adult *Aotus nigriceps* chasing adult *Coendou ichillus*.

**Figure 7.**
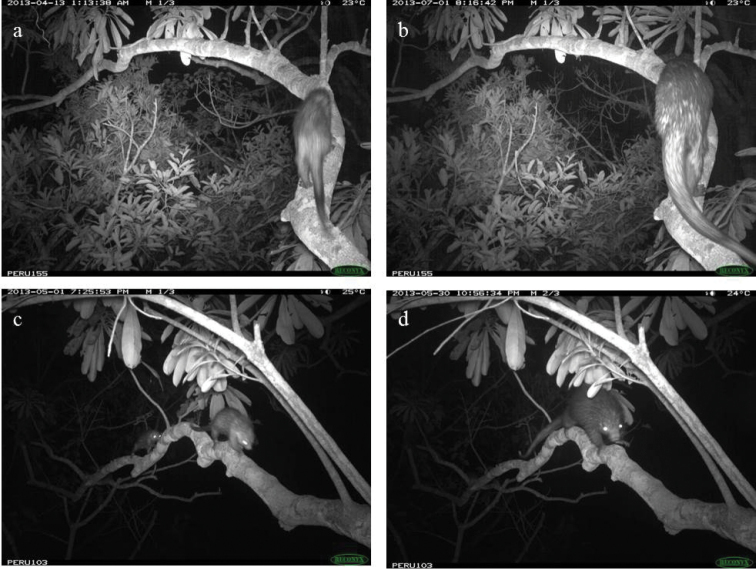
*Coendou ichillus* (**a** and **c**) and *Coendou bicolor* (**b** and **d**) in the same two locations. The species are primarily distinguishable by size differences.

Four interspecific interactions were recorded in three bridges, and they were all with *Aotus nigriceps*. In one of the bridges (N=2 events), an adult *Aotus nigriceps* chased an adult *Coendou ichillus* (Figure 6). *Coendou bicolor* individuals (2 total) were also photographed in two of the same bridges used by *Coendou ichillus* during 20 events (1 event in one bridge and 19 in the other). For five of these events, the two species used the same bridge on the same night. Their extreme difference in body size makes them easily distinguishable (Figure 7).

**Figure 8.**
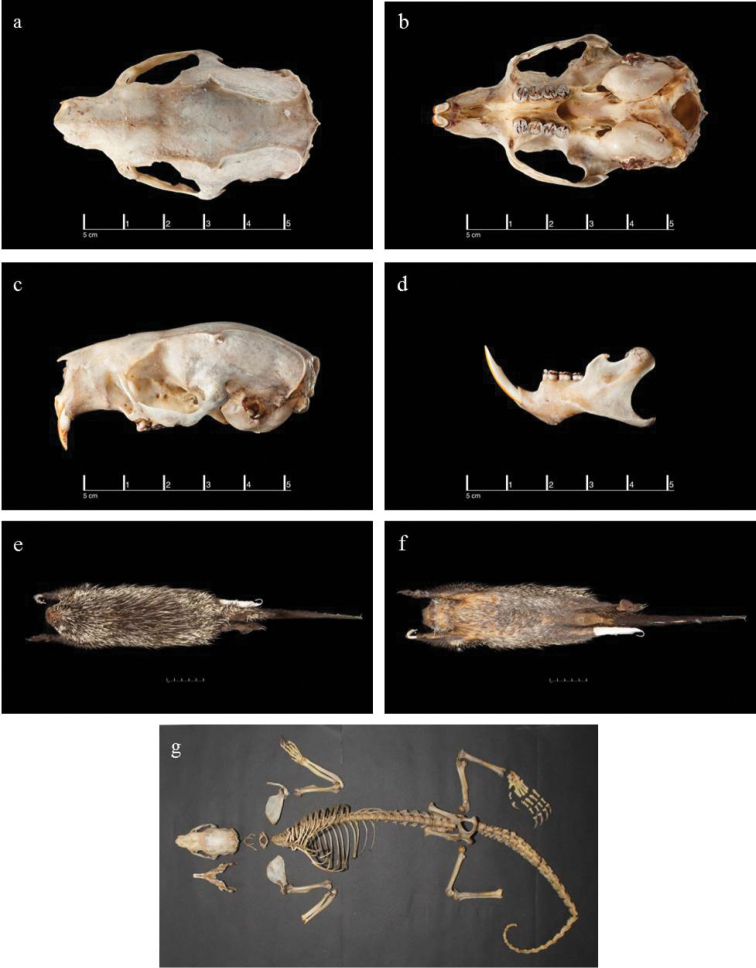
Images of skull (**a** dorsal **b** ventral, and **c** lateral views), mandible (**d**), skin (**e** dorsal and **f** ventral views), and skeleton (**g**) of our *Coendou ichillus* specimen (CORBIDI-MA-00973).

**Figure 9.**
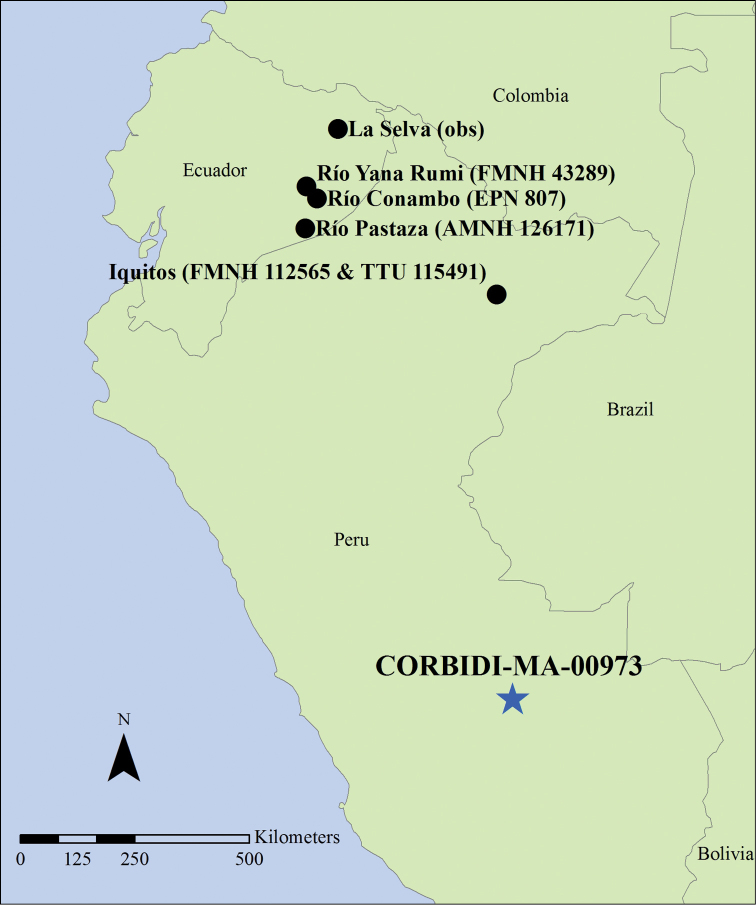
Map of *Coendou ichillus* collection sites and observation sites, including that of the present specimen (blue star) and those of the specimens analyzed by Voss and da Silva (2001), Voss (2011) and Voss et al. (2013) (black circles). Coordinates for all sites, except La Selva (live animal observations) and CORBIDI-MA-00973 are estimated.

Our specimen from the LUR (CORBIDI-MA-00973) fits the description of the holotype of *Coendou ichillus* (Figure 8), as described in Voss and da Silva (2001), and differs only in some details of the pelage as follows: 1) the dorsal view of the skin of the holotype, as photographed in Figure 9 of Voss and Da Silva (2001), shows a distinct, apparently spineless patch over the lower back and on the dorsal surface of the base of the tail, whereas our specimen from Peru is well-quilled over much of this area, there being only a small patch of quill-less skin over the base of the tail that we believe was the result of de-quilling during the skinning of the specimen; 2) Voss and da Silva (2001) describe the dorsal part of the tail of *Coendou ichillus* as having tricolored bristles extending along the lateral caudal surfaces of the tail converging to form an indistinct whitish or yellowish “chevron” near the middle of the tail, but our specimen from the LUR did not exhibit this trait and had a rather sparse covering of tricolored bristles along the sides of the base of the tail; 3) the small tufts of bristles on the outer canthus of the pinnae appear to be set higher on the pinnae compared to those in the animal photographed in figure 12 of Voss and da Silva (2001) such that the ear bristle tufts on our specimen are in a line directly anterior to the eye, whereas in the live animal photographed, these bristles are slightly below an imaginary line drawn posteriorly through the midline of the eye (Figures 2 and 8).

In addition to providing measurements of long bones in Table 1, some additional comments on the overall condition of the bony elements are warranted. The animal appears to have suffered injuries in life that, although healed, are readily apparent on the specimen. There is a healed fracture on the left zygoma, which is slightly indented compared to the normal right side. There are also apparently related distortions to the rostrum, which is somewhat asymmetrical in dorsal view and twisted to the animals’ right side. Finally, the animal appeared to have lost, during its lifetime, the part of the last phalange bearing the claw on the third digit of its left foot. This toe was observed in the field as being clawless but healed over at the time of capture.

**Table 1.** Comparisons of morphometrics of the present specimen of *Coendou ichillus* (CORBIDI-MA-00973) in millimeters with those described by Voss and da Silva (2001, AMNH, EPN, FMNH).

	AMNH	EPN	FMNH	CORBIDI-MA-00973
Sex	unk	female	male	female
**HBL**	ca. 290	-	ca. 260	307
**LT**	ca. 250	-	ca. 210	251
**HF**	-	59	58	56.7
**LA**	-	-	-	22.9
**LA2**	-	-	-	17.6
**CIL**	58.8	64.2	64.4	64.4
**LD**	14	15.8	16.9	17.2
**LIF**	3.7	-	3.7	3.7
**BIF**	2.7	-	3.1	3.5
**MTR**	14.1	15.2	14	14
**LM**	10.3	11.4	10.5	10.4
**BP4**	4.1	4.5	3.9	4.1
**BM1**	3.8	4.2	3.8	3.6
**APB**	4.4	4.5	4.1	4.7
**PPB**	5.9	6.2	6.5	6.5
**PZB**	39.2	39.1	39	38.5
**HIF**	8.6	8.3	9.8	8.2
**ZL**	24	25.7	26.2	24
**LN**	-	18.8	-	19.6
**BNA**	-	9.9	-	9.4
**BB**	29.3	31.3	28.9	29.5
**DI**	2.9	2.9	-	2.9
**BIT**	4.7	4	-	4.5
**Weight**	-	-	-	770g

The vertebral column of our specimen (CORBIDI-MA-00973) presents seven cervical vertebrae, of which C 2-4 were fused. There were 15 thoracic vertebrae with 15 pairs of ribs, six lumbar vertebrae as defined by a conspicuous transverse process, three fused sacral vertebrae, and 29 caudal vertebrae. Greatest lengths of long bones from the left side of the animal are provided in Table 1.

Our specimen represents the sixth known *Coendou ichillus* voucher, and it substantially extends the known range of the species. All previous unambiguous records of the species were from the Amazonian lowlands of eastern Ecuador and Iquitos, Peru (Figure 9), our new record extends the range of the species approximately 900 km further south into the Peruvian Amazon. This unambiguous record of *Coendou ichillus* from the Lower Urubamba Region of Peru confirms that this species has a much wider distribution outside of eastern Ecuador and northeastern Peru.

## References

[B1] Comiskey JA Campbell JP Alonso A Mistry S Dallmeier F Núñez P Beltrán H Baldeón S Nauray W de la Colina R Acurio L Udvardy S (2001) The vegetation communities of the Lower Urubamba Region, Peru. In: Alonso A, Dallmeier F, Campbell P (Eds) Urubamba: The Biodiversity of a Peruvian Rain Forest SI/MAB Series #7. Smithsonian Institution Press, Washington, D.C. http://www.gbv.de/dms/goettingen/353020125.pdf

[B2] Gregory T Carrasco-Rueda F Deichmann JL Kolowski J Alonso A (2012) Primates of the Lower Urubamba Region, Peru, with comments on other mammals. Neotropical Primates 19: 16–23. doi: 10.1896/044.019.0103

[B3] Gregory T Carrasco-Rueda F Deichmann JL Kolowski JM Alonso A (2014) Arboreal camera trapping: Taking a proven method to new heights. Methods in Ecology and Evolution 5: 443–451. doi: 10.1111/2041-210X.12177

[B4] Voss RS (2011) Revisionary notes on Neotropical porcupines (Rodentia: Erethizontidae) 3. An annotated checklist of the species of *Coendou* Lacepede, 1799. American Museum Novitates 3720: 1–36. doi: 10.1206/3720.2

[B5] Voss RS da Silva MNF (2001) Revisionary notes on the Neotropical porcupines (Rodentia: Erethizontidae). 2. A review of the *Coendou vestitus* group with descriptions of two new species from Amazonia. American Museum Novitates 3351: 1–36. doi: 10.1206/0003-0082(2001)351<0001:RNONPR>2.0.CO;2

[B6] Voss RS Emmons L (1996) Mammalian diversity in Neotropical lowland rainforests: A preliminary assessment. Bulletin of the American Museum of Natural History, New York 230: 1–115. http://hdl.handle.net/2246/1671

[B7] Voss RS Hubbard C Jansa SA (2013) Phylogenetic relationships of New World porcupines (Rodentia, Erethizontidae): Implications for taxonomy, morphological evolution, and biogeography. American Museum Novitates New York NY 3769: 1–36. doi: 10.1206/3769.2

